# Association of QTc Interval with Risk of Cardiovascular Diseases and Related Vascular Traits: A Prospective and Longitudinal Analysis

**DOI:** 10.5334/gh.533

**Published:** 2020-02-10

**Authors:** Chanjuan Deng, Jingya Niu, Liping Xuan, Wen Zhu, Huajie Dai, Zhiyun Zhao, Mian Li, Jieli Lu, Yu Xu, Yuhong Chen, Weiqing Wang, Guang Ning, Yufang Bi, Min Xu, Tiange Wang

**Affiliations:** 1 Shanghai National Clinical Research Center for Endocrine and Metabolic Diseases, Key Laboratory for Endocrine and Metabolic Diseases of the National Health Commission of the PR China, Shanghai Institute of Endocrine and Metabolic Diseases, Ruijin Hospital, Shanghai Jiao Tong University School of Medicine, Shanghai, CH

**Keywords:** QTc interval, cardiovascular diseases, vascular traits

## Abstract

**Background:**

Prolonged heart rate corrected QT (QTc) interval was reported to be associated with cardiovascular diseases (CVDs).

**Objective:**

There exists little data on the association between QTc interval and cardiovascular risk in Asian populations. We prospectively investigated the association of QTc interval with CVDs and vascular traits in a large cohort of Chinese adults.

**Methods:**

A total of 7,605 participants aged 40 years or older from a well-defined community without CVDs at baseline were included and followed up for an average of 4.5 years. Association of baseline QTc interval with incident CVDs was evaluated using Cox regression analysis. Associations of QTc interval with brachial-ankle pulse wave velocity (baPWV), carotid intima-media thickness (CIMT), and risk of microalbuminuria and peripheral arterial diseases (PAD) were secondarily examined.

**Results:**

Prolonged QTc interval (≥460 ms in women and ≥450 ms in men) was associated with 51% higher risk of total major CVDs (hazard ratio [HR] = 1.51, 95% confidence interval [CI] [1.20, 1.90]), particularly, 48% increased risk of stroke (95% CI [1.16, 1.88]). Prolonged QTc interval was positively associated with baPWV (β = 38.10 cm/s, standard error [SE] = 8.04, *P* < 0.0001) and CIMT (β = 0.01 mm, SE = 0.01, *P* = 0.04). Prolonged QTc interval was associated with increased risk of incident microalbuminuria (odds ratio [OR] = 1.65, 95% CI [1.21, 2.24]) and PAD (2.49, 95% CI [1.35, 4.59]).

**Conclusions:**

Prolonged QTc interval is positively and significantly associated with increased risk of CVDs and related vascular traits in Chinese population.

## Introduction

Cardiovascular diseases (CVDs) are the leading cause of mortality worldwide, including developing countries such as China, especially with the increasing rates of obesity and Type 2 diabetes, which are the two important risk factors of CVDs [1,2]. It is estimated that in China the number of patients suffering from CVDs is up to 29 million, which will continue growing rapidly in the next 10 years [3]. It is of extreme importance to discover more effective predictors to optimize screening or preventative measures for individuals susceptible to CVDs.

The electrocardiogram (ECG) is a simple, noninvasive diagnostic test to detect functional and structural abnormity of the heart with cardiac electricity activity. The latest recommendation from the American Heart Association (AHA) and the American College of Cardiology Foundation (ACCF) emphasized the diagnostic value of ECG for CVDs in patients with hypertension and diabetes [4]. Of note, QT interval is a common ECG index for diagnosis of ventricular arrhythmia by representing the whole process of electrical repolarization of myocardium [5]. A number of studies have shown that heart rate corrected QT (QTc) interval is strongly associated with the risk of CVDs in different populations [5,6,7,8]. For example, a longitudinal study in Americans identified that QTc interval was an independent predictor of all-cause and cardiovascular mortality in diabetic patients [5]. Another cohort study reported that prolonged QTc interval was associated with a significantly increased risk of stroke in the general population [6]. In addition, QTc interval was demonstrated to improve the accuracy of the personalized CVDs prognosis built on a conventional risk predicting model [9].

Recent studies demonstrated significantly longer QTc intervals of black people compared with those of other races [10,11], indicating a remarkable ethnic and racial variation. The existing studies mostly focused on Western populations; whereas, few studies to date have explored the predictive value of QTc interval for CVDs in East Asians. Furthermore, based on the positive association between prolonged QTc interval and risk of sudden cardiac mortality [10], it is of much significance to detect the prolongation of QTc interval. As such, validation of the association between QTc interval and risk of CVDs in the East Asian population is necessary. Therefore, we performed a prospective analysis to assess the association of QTc interval with the risk of CVDs in a well-defined community-based Chinese cohort; we further performed longitudinal analyses to examine the associations of QTc interval with several CVD-related vascular traits, for instance, the repeated measurements of brachial-ankle pulse wave velocity (baPWV), carotid intima-media thickness (CIMT), and early indicators, urinary albumin excretion, and ankle-brachial index (ABI).

## Methods

### Study population

The prospective analysis was performed in a community-based cohort study that was initiated in 2010 at baseline, Jiading District, Shanghai, China [12,13]. Between March and August 2010, a total of 10,375 residents aged 40 years or older were recruited. For the primary analysis of the present study, we excluded 1,357 participants at baseline for the following reasons: 1) 355 had self-reported CVDs or missed data on CVDs; 2) 879 missed ECG measures; 3) 14 had a cardiac pacemaker placed; and 4) 109 took antiarrhythmic therapy (procainamide, sotalol, flecainide, amiodarone, et al). From August 2014 to May 2015, all 9,018 participants were invited to attend a follow-up visit. After excluding 1,413 individuals who failed to attend the follow-up examination, 7,605 participants were followed up with for the primary analysis of association between QTc interval and incident CVDs. For the secondary analysis, we further excluded 2,654 individuals with missing or poor ECG data, 5 with a cardiac pacemaker, 27 using antiarrhythmic therapy at follow-up, and 667 without complete vascular traits measurements, leaving 4,252 participants for the analysis. Details on the flow chart of study participants are shown in Supplemental Figure 1.

The study protocol was approved by the Institutional Review Board of Ruijin Hospital, Shanghai Jiao Tong University School of Medicine. Written informed consent was obtained from each participant.

### Anthropometric Measurements

Participants were interviewed by trained personnel according to the standard questionnaire involving medical history, medication use, as well as lifestyle factors. The participant was defined as a current smoker if he/she smoked one cigarette per day or seven per week regularly for at least six months. Physical activity was evaluated on the basis of the International Physical Activity Questionnaire (IPAQ) [14]. Metabolic equivalent (MET) was calculated in minutes per week according to IPAQ, and the level of physical activity was divided into three groups: mild (≤599 MET-min/week), moderate (600–2,999 MET-min/week), vigorous (≥3000 MET-min/week) [15,16]. Education attainment was categorized as high school or above and less than high school. Body height and weight were measured by the same trained physicians. Body mass index (BMI) was calculated as body weight in kilograms divided by height squared in meters (kg/m^2^). Systolic blood pressure (SBP) and diastolic blood pressure (DBP) were measured with an automated electronic device (OMRON Model HEM-752 FUZZY, Omron Company, Dalian, China) on the nondominant arm three times consecutively with one-minute intervals after at least 10-minutes’ rest. The average value of the three measurements was adopted in our analysis. Hypertension was defined as SBP ≥ 140 mmHg or DBP ≥ 90 mmHg or physician-diagnosed hypertension and use of antihypertensive medications.

### Laboratory Measurements

All participants underwent a 75-g oral glucose tolerance test (OGTT), and 0 and 2 hours blood samples were collected. The fasting blood glucose (FBG) and 2-hour post-loading blood glucose (PBG) were measured using the glucose oxidize method on an autoanalyzer (Modular P800; Roche, Basel, Switzerland). Diabetes was defined as FPG ≥ 7.0 mmol/L or OGTT-2h PBG ≥ 11.1 mmol/L or self-reported physician-diagnosed diabetes and use of anti-diabetic agents [17]. Fasting serum triglycerides (TG), total cholesterol (TC), high density lipoprotein cholesterol (HDL-C), and low density lipoprotein cholesterol (LDL-C) were determined by the chemiluminescence method using an autoanalyzer (Modular E170; Roche, Basel, Switzerland). Urinary albumin and creatinine concentrations were determined using the first void sterile urine sample in the early morning by rate nephelometry (Beckman Coulter, Fullerton, CA) and alkaline nitroxanthic acid method, respectively [18]. Urinary albumin-to-creatinine ratio (UACR) was calculated by dividing the urinary albumin concentrations by the urinary creatinine concentrations and expressed in mg/g. Microalbuminuria was defined as 30 mg/g ≤ UACR < 300 mg/g [19].

### ECG measures

Participants underwent resting ECG recording with a 12-lead electrocardiograph instrument (CAM14, GE, US). The QT duration was determined from the start of the QRS complex until the end of the T-wave, measured and recorded by the electrocardiograph automatically. To adjust for heart rate, we used Bazett formula: QTc_Baz_ = QT (heart rate/60)^1/2^, where QTc value of ≥460 ms in women and ≥450 ms in men was considered as prolonged QTc interval [20].

### Ascertainment of outcomes and vascular traits

The primary outcome of the current analysis was the composite of incident fatal and nonfatal CVDs, including cardiovascular mortality, myocardial infarction, and stroke. We collected the disease and mortality information on vital status from the local municipal health authorities. For each deceased participant, the date and mostly likely cause of mortality were obtained, and the length of follow-up was determined from the date of first visit in 2010 to the date of mortality. For the other participants, the dates and types of major events were recorded if they happened, and the lengths of follow-up were determined from the dates of first visit in 2010 to the dates of second visit.

For the secondary analyses, the baPWV and ABI were measured by a fully automatic arteriosclerosis diagnosis device (Colin VP-1000, Model BP203RPE II, form PWV/ABI, Japan) with the participants after at least 10-minutes’ rest. Peripheral vascular disease (PAD) was defined as narrowing and obstruction of antegrade flow of major systemic arteries [21], which was diagnosed as ABI < 0.9 or >1.4 at either side [22]. CIMT measurements were performed using a high-resolution B-mode tomographic ultrasound system (Esaote Biomedica SpA, Genoa, Italy) equipped with 7.5 MHz line transducer [23]. The same experienced physician measured CIMT on the far wall of the right and left common carotid arteries, 1.5 cm proximal to the bifurcation.

### Statistical Analysis

Baseline characteristics of the participants were described as the mean (standard deviation, SD) for continuous variables that were normally distributed, the median (interquartile range) for continuous variables of skew distribution, and the frequency (percentage) for categorical variables. Differences between participants with normal QTc interval and those with prolonged QTc interval were examined by unpaired t (for continuous variables) and chi-square (for categorical variables) tests.

Association between QTc interval and incident CVDs was assessed by multivariable Cox regression analysis with QTc interval in per 1-SD increase, as well as prolonged versus normal QTc interval. Models of statistical analysis were performed as follows: model 1, unadjusted; model 2, adjusted for age, sex; model 3, additionally adjusted for BMI, education, current smoking, physical activity, diabetes, hypertension, TC, TG, HDL-C, and LDL-C. We also performed stratified analyses by sex (men vs. women), age (≥median of 57.4 vs. <57.4 years), BMI (≥25 vs. <25 kg/m^2^), high school education or above (yes vs. no), current smoking (yes vs. no), physical activity (mild vs. moderate vs. vigorous), diabetes (yes vs. no) and hypertension (yes vs. no). We tested the multiplicative interactions of QTc interval with stratification factors.

Secondary analyses included 1) examining the association of QTc interval with repeated baPWV and CIMT measurements by generalized estimating equations (GEE); 2) examining the association of QTc interval with newly-developed microalbuminuria and PAD by multivariable logistic regression analysis.

Statistical significance was accepted at a two-sided P value <0.05. All analyses were performed using SAS software, version 9.4 (SAS Institute, Cary, NC).

## Results

The baseline characteristics of the study participants are shown in Table 1. At baseline, 1,137 of the participants (15%) had prolonged QTc interval. Compared with the participants who had normal QTc interval, those with prolonged QTc interval were much older and had higher BMI levels. The prevalence of diabetes and hypertension were also higher in the group of prolonged QTc interval.

**Table 1:** Baseline characteristics of the participants.

Characteristics	Overall(n = 7605)	Normal QTc interval(n = 6468)	Prolonged QTc interval(n = 1137)	*P* value

Men, n (%)	2861 (37.6)	2460 (38.0)	401 (35.3)	0.08
Age, years	58.1 (9.3)	57.6 (9.2)	61.1 (9.8)	0.001
Body mass index, kg/m^2^	25.2 (3.2)	25.1 (3.2)	25.7 (3.5)	0.002
High school education or above, n (%)	1552 (20.4)	1354 (20.9)	198 (17.4)	0.007
Current smoking, n (%)	1565 (21.2)	1368 (21.8)	197 (17.9)	0.004
Physical activity, mild/moderate/vigorous (%)	65.5/21.3/13.2	64.4/21.5/14.1	71.4/20.3/8.3	<0.0001
Systolic blood pressure, mmHg	141.1 (19.9)	139.8 (19.4)	148.7 (21.1)	0.0001
Diastolic blood pressure, mmHg	82.9 (10.4)	82.5 (10.1)	85.2(11.2)	<0.0001
Fasting blood glucose, mmol/L	5.6 (1.5)	5.5 (1.4)	5.9 (2.0)	<0.0001
OGTT-2h blood glucose, mmol/L	8.2 (4.3)	8.0 (4.1)	9.5 (5.2)	<0.0001
Serum triglyceride, mmol/L	1.4 (1.0, 1.9)	1.3 (1.0, 1.9)	1.6 (1.1, 2.2)	0.11
Serum total cholesterol, mmol/L	5.4 (1.0)	5.3 (1.0)	5.5 (1.0)	0.50
Serum HDL cholesterol, mmol/L	1.3 (0.3)	1.3 (0.3)	1.3 (0.3)	0.27
Serum LDL cholesterol, mmol/L	3.2 (0.9)	3.2 (0.9)	3.2 (0.9)	0.81
Diabetes, n (%)	1359 (17.9)	1047 (16.2)	312 (27.4)	<0.0001
Hypertension, n (%)	4565 (60.1)	3716 (57.5)	849 (74.7)	<0.0001
Heart rate, bpm	76.3 (0.9)	76.2 (11.8)	76.7 (12.3)	0.36
QT interval, ms	387.2 (31.7)	383.1 (27.2)	410.4 (43.4)	<0.0001
QTc interval, ms	432.7 (30.2)	424.6 (19.8)	479.3 (36.2)	<0.0001

Data are presented as mean (SD), median (inter-quartile ranges), or proportions. Linear regression for continuous variables and Cochran Armitage trend chi-square test for categorical variables were applied to analyze the difference according to whether prolonged QTc interval happen. OGTT: oral glucose tolerance test; HDL cholesterol: high density lipoprotein cholesterol; LDL cholesterol: low density lipoprotein cholesterol; QTc interval: corrected QT for heart rate using Bazett’s formula [QTc-Baz = QT (heart rate/60)^1/2^]; prolonged QTc interval: QTc interval ≥450 ms in men or QTc interval ≥460 ms in women.

During an average of 4.5 years of follow-up, we documented 419 events of CVDs, including 385 cases of non-fatal and fatal stroke and 36 cases of non-fatal and fatal myocardial infraction; of these cases, the number of cardiovascular mortality was 37. Participants may have experienced more than 1 CVD event.

### Association of QTc interval with risk of incident CVDs

As shown in Table 2, compared with normal QTc interval, prolonged QTc interval was associated with a 51% higher risk of incident CVDs (hazard ratio [HR] = 1.51, 95% confidence interval [CI]: [1.20, 1.90]; *P* = 0.0004); and 48% increased risk of incident stroke (HR = 1.48, 95%CI [1.16, 1.88]; *P* = 0.002), after adjustments for the potential confounding factors (Model 3). We did not find a significant association between prolonged QTc interval and myocardial infarction (HR = 1.52, 95%CI [0.73, 3.17]; *P* = 0.26). The continuous variable analyses with each 1-SD increase in QTc interval as an exposure were also performed. The results showed similar trends but not significant association with CVDs or the specific CVD (Table 2).

**Table 2:** Association between QTc interval and cardiovascular diseases, stroke, and myocardial infraction in Chinese population.

	Events(n, %)	Incidence rates(per 1000 PYs)	QTc interval(per 1-SD increase)*		QTc interval(prolonged^†^ vs. normal)	

			HR (95% CI)	*P* value	HR (95% CI)	*P* value

Cardiovascular diseases	419 (5.5)	12.0				
Model 1			1.17 (1.09, 1.26)	<0.0001	1.99 (1.60, 2.48)	<0.0001
Model 2			1.09 (1.01,1.19)	0.04	1.62 (1.30, 2.03)	<0.0001
Model 3			1.07 (0.98, 1.17)	0.13	1.51 (1.20, 1.90)	0.0004
Stroke	385 (5.1)	11.0				
Model 1			1.16 (1.07, 1.25)	0.0002	1.92 (1.52, 2.42)	<0.0001
Model 2			1.08 (0.99, 1.18)	0.09	1.57 (1.25, 1.99)	0.0001
Model 3			1.06 (0.97, 1.16)	0.23	1.48 (1.16, 1.88)	0.002
Myocardial infraction	36 (0.5)	1.0				
Model 1			1.24 (1.00, 1.53)	0.048	2.50 (1.23, 5.07)	0.01
Model 2			1.20 (0.93, 1.53)	0.16	1.84 (0.89, 3.80)	0.10
Model 3			1.13 (0.86, 1.48)	0.37	1.52 (0.73, 3.17)	0.26

Data are presented as hazard ratio (HR) and 95% confidence interval (CI). *Corrected QT for heart rate using Bazett’s formula (QTc-Baz = QT [heart rate/60]^1/2^), 1-SD: standard deviation, 30.2 ms; ^†^Prolonged QTc interval: QTc interval ≥ 450 ms in men or QTc interval ≥ 460 ms in women; PY: person years. Model 1, unadjusted; Model 2, adjusted for sex, age; Model 3, further adjusted for BMI, education, current smoking, physical activity, diabetes, hypertension, serum total cholesterol, triglycerides, HDL cholesterol and LDL cholesterol based on model 2. BMI: body mass index; HDL cholesterol: high density lipoprotein cholesterol; LDL cholesterol: low density lipoprotein cholesterol.

Supplemental Figure 2 shows the multivariable-adjusted HRs for CVDs and stroke in relation to prolonged versus normal QTc interval, in subgroups stratified by sex (men vs. women), age (≥median of 57.4 vs. <57.4 years), BMI (≥25 vs. <25 kg/m^2^), high school education or above (yes vs. no), current smoking (yes vs. no), physical activity (mild vs. moderate vs. vigorous), diabetes (yes vs. no), and hypertension (yes vs. no). It is shown that prolonged QTc interval (vs. normal) was significantly associated with an increased risk of CVDs across most subgroups, except for that in participants younger than median age of 57.4 years, current smokers, those with high school education or above, moderate physical activity, and those without hypertension (Supplemental Figure 2A). The interactions between QTc interval and stratification factors were not significant (all *P* > 0.05). Similar results were observed in the subgroup analysis when we analyzed the association of prolonged QTc interval with stroke (Supplemental Figure 2B).

### Association of QTc interval with vascular traits using repeated measurements

As shown in Table 3, the average value of baPWV was 1585 ± 345 cm/s at baseline and 1661 ± 344 cm/s at follow-up. The corresponding value of CIMT was 0.6 ± 0.1 mm and 0.7 ± 0.2 mm. The repeated measures analysis revealed that QTc interval was positively associated with the increase in baPWV and CIMT, after adjustments for sex, age, BMI, current smoking, physical activity, education, diabetes, hypertension, TC, TG, HDL-C, and LDL-C (baPWV: β = 0.56 cm/s, Standard error [SE] = 0.11, *P* < 0.0001; CIMT: β = 0.0001 mm, SE = 0.0001, *P* = 0.03). When we regarded QTc interval as a categorical variable, similar results were shown (prolonged vs normal QTc interval: baPWV: β =38.10 cm/s, SE = 8.04, *P* < 0.0001; CIMT: β = 0.01 mm, SE = 0.01, *P* = 0.04).

**Table 3:** Association of QTc interval with vascular markers using repeated measurements.

	Baseline	Follow-up	QTc interval(per 1-ms increase)		QTc interval(prolonged* vs. normal)	

			β (SE)	*P* value	β (SE)	*P* value

baPWV, cm/s	1585 (345)	1661 (344)	0.56 (0.11)	<0.0001	38.10 (8.04)	<0.0001
CIMT, mm	0.6 (0.1)	0.7 (0.2)	0.0001 (0.0001)	0.03	0.01 (0.01)	0.04

Parameter estimates were computed from separate generalized estimating equation analysis model with each variable at a time after adjustment for sex, age, BMI, current smoking, physical activity, education, diabetes, hypertension, serum total cholesterol, triglycerides, HDL cholesterol and LDL cholesterol. *Prolonged QTc interval: QTc interval ≥ 450 ms in men or QTc interval ≥ 460 ms in women; baPWV: brachial-ankle pulse wave velocity; CIMT: carotid intima-media thickness; SE: standard error.

### Association of QTc interval with early vascular diseases

**Figure 1:**
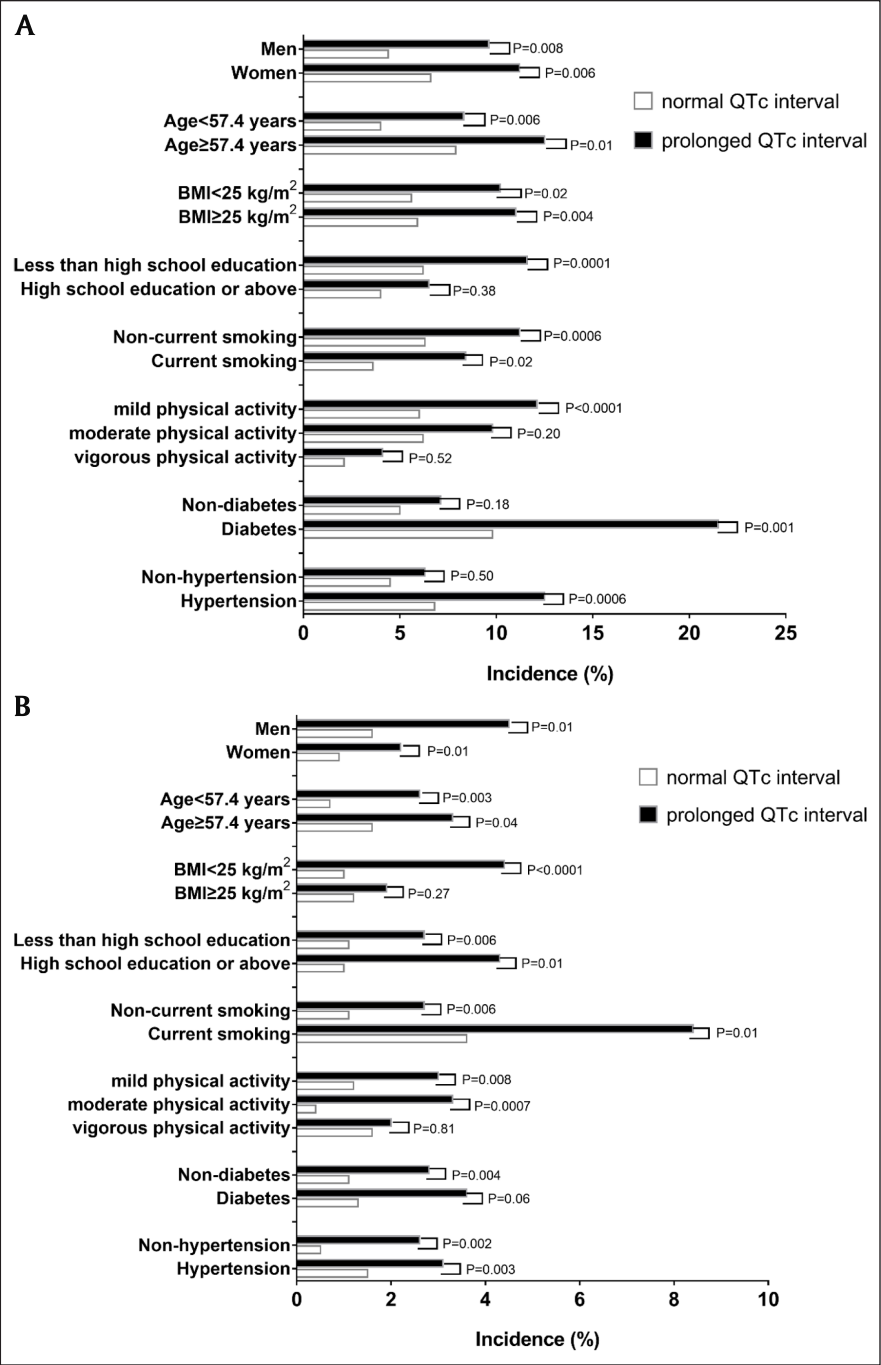
Incidences of microalbuminuria **(A)** and PAD **(B)** according to subgroups, comparing the prolonged QTc interval group with the normal QTc interval group. Prolonged QTc interval: QTc interval ≥ 450 ms in men or QTc interval ≥ 460 ms in women; PAD: peripheral arterial disease.

When examining the association of baseline QTc interval with newly developed microalbuminuria and PAD, we additionally excluded a corresponding 267 and 290 individuals because of suffering from the respective disease at baseline in 2010. Figure 1 and Supplemental Table 1 show incidences of microalbuminuria and PAD according to subgroups, comparing the prolonged QTc interval group with the normal QTc interval group. Compared with the group of normal QTc interval, the group of prolonged QTc interval had higher incidences of microalbuminuria and PAD across all the subgroups (Figure 1). In the multivariable logistic regression analysis, we found that prolonged QTc interval was associated with an increased risk of newly developed microalbuminuria (odds ratio [OR] = 1.65, 95% CI [1.21, 2.24]; *P* = 0.002) and PAD (2.49, 95% CI [1.35, 4.59]; *P* = 0.004), after adjustments for sex, age, BMI, current smoking, physical activity, education, diabetes, hypertension, TC, TG, HDL-C, and LDL-C. Incidences of microalbuminuria and PAD comparing the prolonged QTc interval group with the normal QTc interval group are shown in Supplemental Figure 3. Compared with the group of normal QTc interval, the group of prolonged QTc interval had a higher incidence of microalbuminuria (10.7% vs. 5.9%, *P* < 0.0001) and PAD (3.0% vs. 1.1%, *P* = 0.0004).

## Discussion

In this cohort study in a general population aged 40 and above, prolonged QTc interval was significantly associated with increased risks of incident CVDs and stroke. In addition, prolonged QTc interval was associated with the increases in baPWV, CIMT in repeated measurements analysis, and increased risks of newly developed vascular diseases, microalbuminuria, and PAD.

The QT interval in the surface electrocardiogram is the total duration of ventricular depolarization and repolarization [24]. As an electrophysiological disorder, prolonged QTc interval often occurs when ventricular repolarization is lengthened, consequently resulting in malignant ventricular arrhythmias [24,25]. A number of cohort studies have shown that prolonged QTc interval may act as a predictor for CVDs in Western populations [11,26]. However, the prevalence of QTc interval prolongation is not consistent in various ethnicities. For example, the prevalence of QTc interval prolongation is below 5% among Americans [27,28] but is up to 13.7% in Chinese [29], suggesting a higher presence of prolonged QTc interval in Chinese than those in other populations. In our study based on participants aged 40 and above, the prevalence of prolonged QTc interval was nearly 15%. It has been reported that there could be a great variation of QTc interval among different ethnic populations caused by genetic risk factors [11,30,31]. A multi-ethnic study of Europeans and Japanese found that 28% of acquired long QT syndrome subjects had mutations in congenital long QT syndrome genes, which differed among races [32]. Although the evidence of QT-related genetic variation in the Chinese population is scarce, the data from a Japanese population have shown a markedly higher prevalence of genetic mutation carriers associated with prolonged QTc interval than in Western populations [32]. In accordance with previous studies, our findings confirmed the association of QTc interval with CVDs by expanding the evidence to the general population but not only limited to the Type 2 diabetic patients [5]. We did not detect a significant association between QTc interval and myocardial infarction in the present study, which might be due to a relatively low incidence and less power to get a statistical significance. In multivariable-adjusted stratification analysis, although the association of prolonged QTc interval (vs. normal) with CVDs appeared to be more pronounced among non-current smokers than among current smokers, the association pattern was consistent between current smokers and non-current smokers, confirmed by a nonsignificant interaction between QTc interval and smoking status (P for interaction = 0.86; Supplementary Figure 2).

QTc interval has been reported as a novel risk factor for progression of albuminuria in patients with Type 2 diabetes [33]. Our study also verified the association between QTc interval and microalbuminuria in the general population, which adjusted the confounding effect of Type 2 diabetes. Consistent with the Hisayama Study among the general population of Japan [34], we found QTc interval prolongation was independently associated with the increase of baPWV. We analyzed two repeated measurement data at both baseline and follow-up to correct random error from measurements or population and to provide more reliable evidence supporting the association. For the first time, we found that prolonged QTc interval was associated with the increase in CIMT and an increased risk of PAD, indicating that QTc interval prolongation could reflect a deterioration of vascular health condition.

The mechanisms underlying the observed associations of QTc interval with CVDs are not quite clear. However, several physiological processes involved as reported before may help interpret the observed association of QTc interval with CVDs in the study. Inflammation, one of the well-established traditional cardiovascular risk factors, can cause widespread endothelial dysfunction, increase oxidative stress, and reduce vascular nitric oxide (NO) bioavailability [35]. The decrease in the content of NO could inhibit the activity of Ca^2+^-ATPase and K^+^/Na^+^-ATPase, thus leading to an increase of cytosolic free calcium and a delay of myocardial repolarization [36]. In addition, periphery blood influx could be accelerated and the ventricular load could be gradually increased as atherosclerosis were intensified by erosions of long-term cardiovascular inflammations and oxidative stress [37]. In this notorious pathogenesis of CVDs, prolongation of the QTc interval could be promoted by myocardial and electrophysiological remodeling [38].

The strengths of this study included a well-defined community-based cohort study, a relatively large sample size, and repeated measurements to evaluate multiple early vascular traits, including baPWV, ABI, CIMT, and urinary albumin excretion. In addition, we collected information on CVDs from local municipal health authorities, which was sufficiently validated, and adjusted fully well-defined confounding factors to reach a reliable conclusion. We also adopted multiple vascular measurements to comprehensively interpret the underlying association of QTc interval with the conditions of vessels. Meanwhile, by GEE, we analyzed repeated measurements at both baseline and follow-up to reduce random errors and to improve reliability for the results. Based on the robust and consistent results, we could convincingly conclude that prolonged QTc interval was associated with an increased risk of CVDs in a Chinese population.

### Study limitations

This study has several notable limitations. First, the Bazett formula, which we used in the study to calculate heart rate corrected QT interval, may overcorrect the impact of heart rate when it is faster than 100 bpm [39]. However, it has been validated and commonly used in clinical practice and epidemiological investigations [27]. Second, because we only had two measurements of QTc interval based on baseline and follow-up, we could not assess the association between the variation of QTc interval during repeated follow-up periods and the risk of CVDs, which required data from subsequent follow-ups. Third, our study was restricted to middle-aged and elderly adults who were mostly Han Chinese, making it inappropriate to generalize our conclusions to younger populations or other ethnic populations.

## Conclusions

Our study demonstrated positive associations of QTc interval with CVDs and several vascular traits as well as early vascular diseases. Given a high prevalence of prolongation of QTc interval in the Chinese population, our findings suggest that the prolongation of QTc interval could be applied in the early identification and prevention of CVDs in middle aged and elderly populations.

## Additional Files

The additional files for this article can be found as follows:

10.5334/gh.533.s1 Supplemental Table 1. Incidences of microalbuminuria and PAD according to subgroups, comparing the prolonged QTc interval group with the normal QTc interval group.

10.5334/gh.533.s2 Supplemental Figure 1. Flow chart of study population.

10.5334/gh.533.s3 Supplemental Figure 2. Hazard ratios and 95% confidence intervals of cardiovascular diseases (A) and stroke (B) according to subgroups, comparing the prolonged QTc interval group with the normal QTc interval group.

10.5334/gh.533.s4 Supplemental Figure 3. Incidences of microalbuminuria (A) and PAD (B) comparing the prolonged QTc interval group with the normal QTc interval group.
