# Supplementary material for: Association of QTc Interval with Risk of Cardiovascular Diseases and Related Vascular Traits: A Prospective and Longitudinal Analysis
Source: Glob Heart. 2020 Feb 10;15(1):13. doi: 10.5334/gh.533 (PMC7218767; doi:10.5334/gh.533)
Supplement: Supplemental Table 1. — Incidences of microalbuminuria and PAD according to subgroups, comparing the prolonged QTc interval group with the normal QTc interval group. [file gh-15-1-533-s1.pdf]

Supplemental Table 1. Incidences of microalbuminuria and PAD according to subgroups, comparing the prolonged QTc interval group with the normal QTc interval group

	Microalbuminuria (n, %)		PAD (n, %)	
	Prolonged QTc interval	Normal QTc interval	Prolonged QTc interval	Normal QTc interval
Sex				
Men	18 (9.6)	57 (4.4)	8 (4.5)	20 (1.6)
Women	40 (11.2)	142 (6.6)	8 (2.2)	17 (0.8)
Age				
Age<57.4 years	19 (8.3)	78 (4.0)	6 (2.6)	13 (0.7)
Age≥57.4 years	39 (12.5)	121 (7.9)	10 (3.3)	24 (1.6)
BMI				
BMI<25 kg/m^2	23 (10.2)	98 (5.6)	10 (4.4)	17 (1.0)
BMI≥25 kg/m^2	35 (11.0)	101 (5.9)	6 (1.9)	20 (1.2)
High school education				
No	52 (11.6)	170 (6.2)	12 (2.7)	30 (1.1)
Yes	6 (6.5)	29 (4.0)	4 (4.3)	7 (1.0)
Current smoking				
No	50 (11.2)	174 (6.3)	12 (2.7)	30 (1.1)
Yes	8 (8.4)	25 (3.6)	4 (4.3)	7 (1.0)
Physical activity				
Mild	45 (12.1)	129 (6.0)	11 (3.0)	26 (1.2)
Moderate	11 (9.8)	49 (6.2)	4 (3.3)	3 (0.4)
Vigorous	2 (4.1)	21 (2.1)	1 (2.0)	8 (1.6)
Diabetes				
No	29 (7.1)	145 (5.0)	11 (2.8)	30 (1.1)
Yes	29 (21.5)	54 (9.8)	5 (3.6)	7 (1.3)
Hypertension				
No	10 (6.3)	68 (4.5)	4 (2.6)	7 (0.5)
Yes	48 (12.5)	131 (6.8)	12 (3.1)	30 (1.5)

Prolonged QTc interval: QTc interval ≥ 450 ms in men or QTc interval ≥ 460 ms in women.PAD: peripheral arterial disease; BMI: body mass index
