# Supplementary figures and images for: Association of QTc Interval with Risk of Cardiovascular Diseases and Related Vascular Traits: A Prospective and Longitudinal Analysis
Source: Glob Heart. 2020 Feb 10;15(1):13. doi: 10.5334/gh.533 (PMC7218767; doi:10.5334/gh.533)

**Supplemental Figure 1. Flow chart of study population**

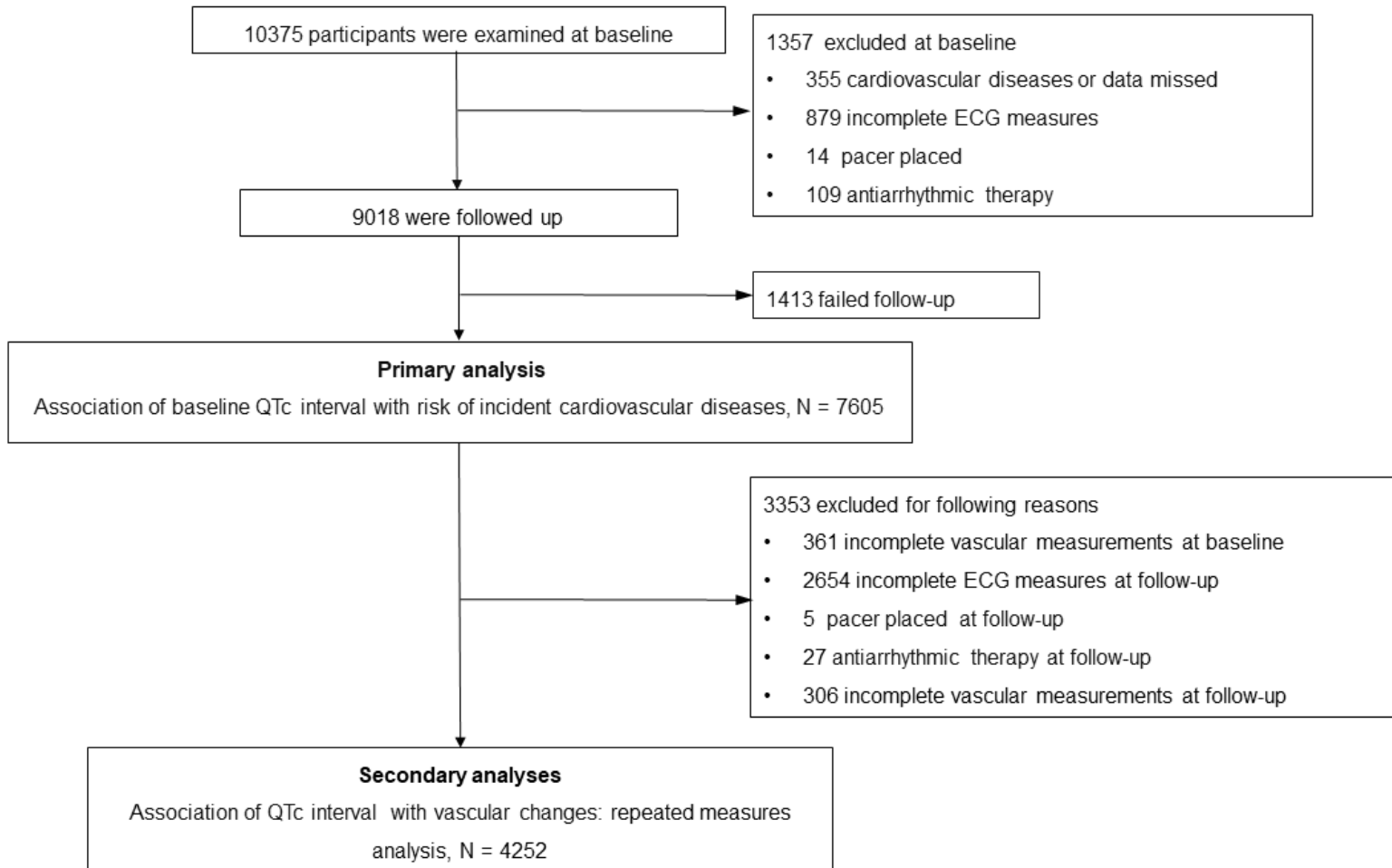

Supplement: Supplemental Figure 1. — Flow chart of study population. [file gh-15-1-533-s2.pdf]
