# Supplementary material for: Association of QTc Interval with Risk of Cardiovascular Diseases and Related Vascular Traits: A Prospective and Longitudinal Analysis
Source: Glob Heart. 2020 Feb 10;15(1):13. doi: 10.5334/gh.533 (PMC7218767; doi:10.5334/gh.533)
Supplement: Supplemental Figure 2. — Hazard ratios and 95% confidence intervals of cardiovascular diseases (A) and stroke (B) according to subgroups, comparing the prolonged QTc interval group with the normal QTc interval group. [file gh-15-1-533-s3.pdf]

Supplemental Figure 2. Hazard ratios and 95% confidence intervals of cardiovascular diseases (A) and stroke (B) according to subgroups, comparing the prolonged QTc interval group with the normal QTc interval group. Hazard ratios (HR) and 95% confidence intervals (CI) were obtained from multiple Cox regression models, after adjustments for sex, age, BMI, education, current smoking, physical activity, diabetes, hypertension, serum total cholesterol, triglycerides, HDL cholesterol and LDL cholesterol; prolonged QTc interval: QTc interval ≥ 450 ms in men or QTc interval≥ 460 ms in women; BMI: body mass index; HDL cholesterol: high density lipoprotein cholesterol; LDL cholesterol: low density lipoprotein cholesterol.

A

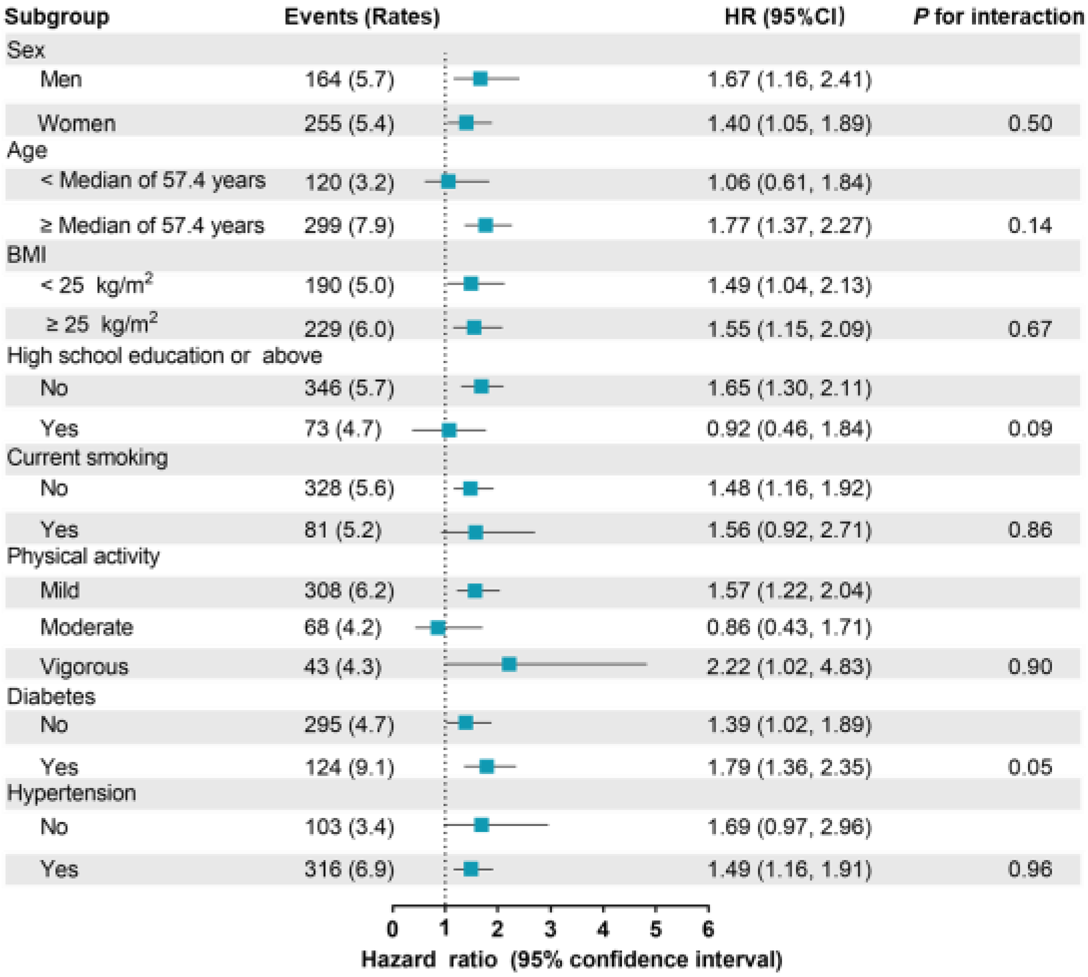

B

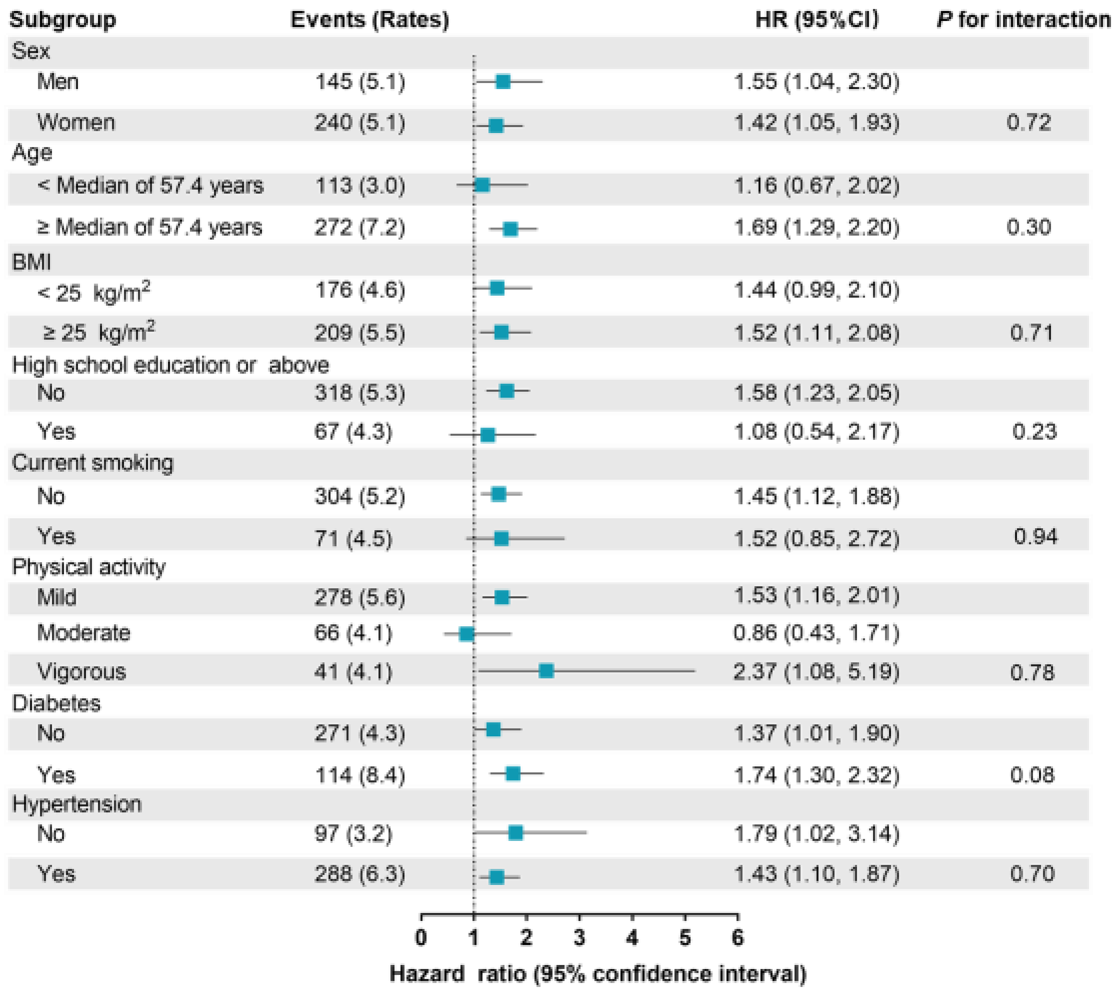
Subgroup
Events (Rates)
HR (95%CI)
P for interaction
Sex
Men 145 (5.1) 1.55 (1.04, 2.30)
Women 240 (5.1) 1.42 (1.05, 1.93) 0.72
Age
< Median of 57.4 years 113 (3.0) 1.16 (0.67, 2.02)
≥ Median of 57.4 years 272 (7.2) 1.69 (1.29, 2.20) 0.30
BMI
< 25 kg/m2 176 (4.6) 1.44 (0.99, 2.10)
≥ 25 kg/m2 209 (5.5) 1.52 (1.11, 2.08) 0.71
High school education or above
No 318 (5.3) 1.58 (1.23, 2.05)
Yes 67 (4.3) 1.08 (0.54, 2.17) 0.23
Current smoking
No 304 (5.2) 1.45 (1.12, 1.88)
Yes 71 (4.5) 1.52 (0.85, 2.72) 0.94
Physical activity
Mild 278 (5.6) 1.53 (1.16, 2.01)
Moderate 66 (4.1) 0.86 (0.43, 1.71)
Vigorous 41 (4.1) 2.37 (1.08, 5.19) 0.78
Diabetes
No 271 (4.3) 1.37 (1.01, 1.90)
Yes 114 (8.4) 1.74 (1.30, 2.32) 0.08
Hypertension
No 97 (3.2) 1.79 (1.02, 3.14)
Yes 288 (6.3) 1.43 (1.10, 1.87) 0.70
0 1 2 3 4 5 6
Hazard ratio (95% confidence interval)
