# Supplementary material for: Association of QTc Interval with Risk of Cardiovascular Diseases and Related Vascular Traits: A Prospective and Longitudinal Analysis
Source: Glob Heart. 2020 Feb 10;15(1):13. doi: 10.5334/gh.533 (PMC7218767; doi:10.5334/gh.533)

Supplemental Figure 3. Incidences of microalbuminuria (A) and PAD (B) comparing the prolonged QTc interval group with the normal QTc interval group. Prolonged QTc interval: QTc interval ≥ 450 ms in men or QTc interval ≥ 460 ms in women; PAD: peripheral arterial disease.

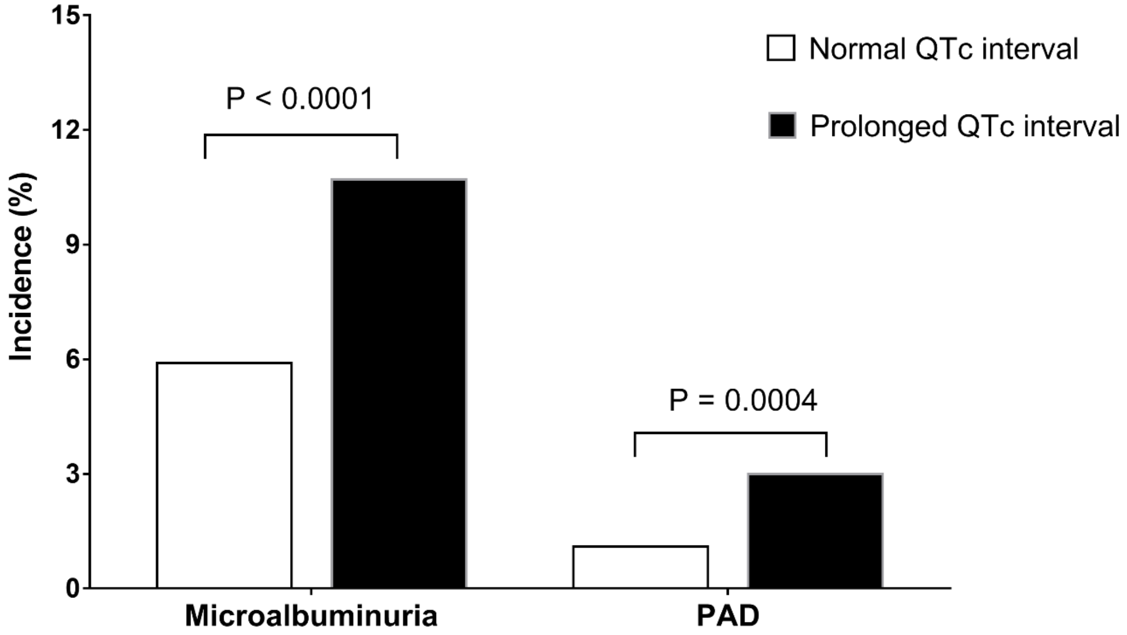

Supplement: Supplemental Figure 3. — Incidences of microalbuminuria (A) and PAD (B) comparing the prolonged QTc interval group with the normal QTc interval group. [file gh-15-1-533-s4.pdf]
